# Genome survey sequencing of the phyto-parasitic nematode *Hoplolaimus galeatus*

**DOI:** 10.7717/peerj.12749

**Published:** 2022-01-18

**Authors:** Xinyuan Ma, Paula Agudelo, Vincent P. Richards, J. Antonio Baeza

**Affiliations:** 1 Department of Biological Sciences, Clemson University, Clemson, SC, United States of America; 2 Plant and Environmental Sciences Department, Clemson University, Clemson, SC, United States of America; 3 Smithsonian Marine Station at Fort Pierce, Smithsonian Institution, Fort Pierce, FL, United States of America; 4 Departamento de Biologia Marina, Universidad Catolica del Norte, Coquimbo, IV Region, Chile

**Keywords:** Nematode, Repetitive elements, Genome, Mitochondrial genome, Genome skimming

## Abstract

**Background:**

*Hoplolaimus galeatus* is a plant-parasite nematode with a broad range of hosts. This nematode is known to damage cotton, corn, and soybean crops. *Hoplolaimus galeatus* is also an economically important pest of turfgrasses. Despite its economical importance, no genomic resources exist for this parasite.

**Methods:**

Using 300 bp paired-end short read sequencing, this study estimated genome size, analyzed a nearly complete mitochondrial chromosome, and explored nuclear repetitive elements, including microsatellites, in *H. galeatus* for the first time. The phylogenetic placement of *H. galeatus* in the superfamily Tylenchoidea was also examined.

**Results:**

The average haploid genome size estimated using a k-mer approach was 517.69 Mbp. The partially assembled mitochondrial genome of *H. galeatus* is 16,578 bp in length and comprised of 11 protein-coding genes, two ribosomal RNA genes, and 16 transfer RNA genes. A maximum likelihood phylogenetic analysis confirmed the monophyly of the genus *Hoplolaimus* and the superfamily Tylenchoidea. Repetitive elements constituted  50% of the nuclear genome while half of the genome represented single- or low-copy sequences. A large portion of repetitive sequences could not be assigned to known repeat element families. Considering only annotated repetitive elements, the most ubiquitous belonged to Class II- Subclass 2-Maverick elements, Class I-LTR-Ty-3/Bel-Pao elements, and satellites. 45S ribosomal DNA was also abundant and a total of 36 SSRs were identified.This study developed genomic resources for the plant-parasitic nematode *Hoplolaimus galeatus* that will contribute to the better understanding of meta-population connectivity and putative genomic mechanisms involved in the exploitation of the broad range of host plants used by *H. galeatus*.

## Introduction

Within the phylum Nematoda, a species-rich clade of ecdysozoan invertebrates (Hodda, 2011), lance nematodes (class Chromadorea, infraorder Tylenchomorphaare) are a monophyletic clade of ecto- and endoparasites that exhibit a distinct cephalic region, a massive well-developed stylet, and infest a wide variety of host plants, including turf grasses, cereals, soybean, corn, cotton, sugar cane, and several trees (Siddiqi, 2000).

Among lance nematodes, *Hoplolaimus galeatus* is a widely distributed species. In the USA, *H. galeatus* can be found along the East Coast from New England to Florida, along the Mississippi River basin (from Minnesota and Wisconsin to Louisiana), in Colorado, Texas, and southern California (Crow & Brammer, 2001; Ye, 2018). This species is also found in Canada, Sumatra, India, Tanzania, as well as in Central and South America (Crow & Brammer, 2001; Ye, 2018). This phyto-pathogen feeds and reproduces on a wide range of host plants, including Bermuda grass, boxwood, Chinese holly, corn, cotton, creeping bentgrass, creeping grasses, slash pine, soybean, tall fescue, and white clover (Ye, 2018). Importantly, *H. galeatus* is known to damage cotton (Gazaway & McLean, 2013), corn (Norton & Hinz, 1976), and soybean crops (Lewis et al., 1993). *Hoplolaimus galeatus* is also an economically important pest of turfgrasses like St. Augustine grass (*Stenotaphrum secundatum*) and bermudagrass (*Cynodon dactylon*) (Ye, 2018). Together with other plant-parasitic nematodes, *H. galeatus* may cause environmental problems indirectly due to the overuse of chemicals during their management in the field (Sher, 1963; Ma et al., 2011; Ma et al., 2019). Despite its economical importance, no genomic resources exist for this nematode pest. The development of such resources is of utmost relevance to improving our understanding not only of the biology of *H. galeatus* but its impact in economically important plants in the USA and beyond, and ultimately, for informing pest management strategies.

This study is part of a comprehensive research program to develop genomic resources in plant-pathogen nematodes from the southeastern USA. Using low-coverage (less than 20×depth) short read next generation sequencing, herein we estimated for the first time the genome size using an *in-silico* k-mer approach, assembled a nearly complete mitochondrial genome with over 200x depth, explored repetitive elements in the nuclear genome, and discovered microsatellites. These newly developed resources will contribute to understanding population connectivity in this pathogen and can also guide the future development of pesticides.

## Materials & Methods

### Sampling and DNA extraction

Turf grass soil samples with specimens of *Hoplolaimus galeatus* were collected from Brightview Landscape, The Villages, Florida (28°58′01.9″N 82°00′02.7″W) and transported to Clemson University Nematode Assay Laboratory for further study. In the laboratory, the sugar centrifugal flotation method was used to extract nematodes from soil samples as previously described (Jenkins, 1964; Handoo & Golden, 1992; Ma et al., 2020). Specifically, a few fixed specimens were identified morphologically referring to diagnostic key characters under the microscope (Handoo & Golden, 1992). For DNA extraction, live nematodes (*n* = 9) were cleaned using distilled water, 3% hydrogen peroxide solution (Aaron Industry, Clinton, SC, USA), DNA Away solution (Aaron Industry, Clinton, SC, USA) and PCR-grade water as described in Ma et al. (2020). DNA extraction followed Ma et al. (2011). Total DNA from each *H. galeatus* specimen was extracted using a Sigma-Aldrich extract-N-Amp kit (XNAT2) (Sigma-Aldrich, St. Louis, MO, USA). Whole-genome amplification (WGA) of a single nematode specimen was then conducted three times using an Illustra Ready-To-Go GenomiPhi V3 DNA amplification kit (GE Healthcare, Chicago, IL, USA) following the manufacturer’s instructions. The highest DNA concentration of the amplification product tested using a Qubit fluorometer (Invitrogen, Carlsbad, CA, USA) was selected for library preparation.

### Library preparation and sequencing

Library preparation and sequencing was conducted as previously described in Ma et al. (2020). Specifically, the Nextera XT kit (Illumina, San Diego, CA, USA) was used for library preparation using the manufacturer’s instructions. Library concentration and fragment size distribution after library preparation were determined using a Qubit fluorometer (Invitrogen, Carlsbad, CA, USA) and a Bioanalyzer 2100 (Agilent Technologies, Santa Clara, CA, USA), respectively. Sequencing was conducted in an Illumina MiSeq with the v3 chemistry kit (Illumina, San Diego, CA, USA). A total of 12,592,874 paired-end (PE) reads (300 bp) were generated and 98.11% of these reads were of high-quality with quality score (*Q*-score) > 30. All raw data are available in the Sequence Read Archive (SRA) repository (BioProject: PRJNA659265, BioSample: SAMN15902603, accession number SRR12516298) at GenBank.

### Genome size estimation using an in-silico k-mer count approach

Raw reads were trimmed using Trimmomatic 0.36 (Bolger, Lohse & Usadel, 2014) to clean adapters. The quality of trimmed reads was then double checked using FastQC (http://www.bioinformatics.babraham.ac.uk/projects/fastqc/), and synchronized using Fastq-pair (Edwards & Edwards, 2019). The totality of the 12,029,684 (6,014,842 pairs) reads was used for the estimation of genome size by counting k-mers with word size = 21 in the software Jellyfish-2 (Marçais & Kingsford, 2011). The k-mer frequency distribution was then processed with the program RESPECT 1.0 (Sarmashghi et al., 2021).

### Mitochondrial genome of *Hoplolaimus galeatus*

The mitochondrial genome of *H. galeatus* was assembled *de novo* using the pipeline GetOrganelle v1.6.4 (Jin et al., 2020). The mitochondrial genome of the congeneric *H. columbus* (available in GenBank: MH657221), was used as a seed. The run used k-mer sizes of 21, 55, 85, and 115. The newly assembled mitochondrial genome was then annotated in the MITOS2 web server (http://mitos.bioinf.uni-leipzig.de/) (Bernt et al., 2013) using the invertebrate genetic code.

### Phylogenetic placement of *Hoplolaimus galeatus*

The phylogenetic position of *Hoplolaimus galeatus* among other representatives of the superfamily Tylenchoidea was explored as previously reported in Ma et al. (2020). Specifically, a total of 13 species belonging to the superfamily Tylenchoidea, including the congeneric *Hoplolaimus columbus*, were included in the phylogenetic analysis. Also, two other species belonging to the class Chromoroidea; *Caenorhabditis elegans* (non-parasitic) and *Ascaris suum* (animal-parasitic), were used as outgroup terminals in our phylogenetic analysis. Each of a total of 12 PCGs was first aligned using MAFFT version 7 (Kuraku et al., 2013) and output files converted into Phylip format using the web server Phylogeny.fr (Dereeper et al., 2008; Dereeper et al., 2010). Then, poorly aligned positions in each of the 12 PCG sequence alignments were trimmed using BMGE (block mapping and gathering with entropy) (Criscuolo & Gribaldo, 2010). The program Sequence Dataset builder (SEDA) (https://www.sing-group.org/seda/index.html) (López-Fernández et al., 2018) was used to concatenate all 12 PCG alignments in the following order: *atp6-cox1-cox2-cox3-cytb-nad1-nad2-nad3-nad4-nad4L-nad5-nad6* (with the exception of *nad4L* missing in *H. galeatus*). The GTR + G nucleotide substitution model selected using SMS (smart model selection) (http://www.atgc-montpellier.fr/sms/) (Lefort, Longueville & Gascuel, 2017) was used for maximum likelihood (ML) phylogenetic analysis conducted on the web server IQ-Tree (http://www.iqtree.org/) (Nguyen et al., 2015) with the default settings but enforcing the GTR + G model of nucleotide substitution. A total of 100 bootstrap replicates were employed to explore support for each node in the resulting phylogenetic tree that was depicted using the web server iTOL (Interactive Tree of Life) (https://itol.embl.de/) (Letunic & Bork, 2019).

### Repetitive elements in the genome of *Hoplolaimus galeatus*

Repetitive elements in the genome of *H. galeatus* were discovered, quantified, and annotated using the pipeline RepeatExplorer 2.3.8 implemented in the platform Galaxy (https://palfinder.ls.manchester.ac.uk/) (Novak et al., 2013) as previously described in Baeza (Baeza, 2021). Specifically, RepeatExplorer uses short sequences randomly sampled from a genome as an input and performs graph-based clustering analysis of sequence read similarities to identify repetitive elements without the need for reference databases of known elements (Novak et al., 2013). RepeatExplorer starts with an all-to-all sequence comparison to find pairs of reads that are similar (90% sequence similarity spanning at least 55% of the read length) and built graph-based clusters of overlapping reads that represent different individual families of repetitive elements. Each of the identified repetitive element clusters is further classified when annotated using an internal data base. Within each cluster, the reads are also assembled into contigs using the program CAP3 (Huang & Madan, 1999) and annotated using the Metazoa version 3.0 repeat dataset included in the package. The genome proportion of each repetitive element cluster was calculated as the percentage of reads (Novak et al., 2013).

### Microsatellite discovery in *Hoplolaimus galeatus*

Simple sequence repeats (SSRs) in the genome of *H. galeatus* were identified using the pipeline Pal_finder v0.02.04.08 as implemented in the platform Galaxy (https://palfinder.ls. manchester.ac.uk) (Griffiths et al., 2016). The pipeline first scanned all short reads for the existence of SSRs (di-, tri-, tetra-, penta-, and hexa-nucleotide motif repeats). Next, PCR primers are developed using default parameters in the software Primer3 (Untergasser et al., 2012). The default settings and most stringent filtering options in pal_filter were applied to select optimal SSR loci; only loci with ’perfect’ motifs, ranked by motif size, and with designed primers were included, and loci where the primer sequences occurred more than once in the set of reads were excluded. A minimum of five repeats were requested for the program pal_finder to select 2-mer SSRs and a minimum of six repeats to select SSRs with three, four, five, and six repeat motifs.

## Results

### Genome size in-silico estimation of *Hoplolaimus galeatus*

The average haploid genome of *H. galeatus* size estimated using a k-mer approach was 417.69 Mbp, with a moderate level of genome heterozygosis (het. = 4.34%) and a relatively low unique genome content (25%). The estimated genome size of *H. galeatus* is relatively large. Furthermore, together with the abundance of repetitive elements (see below), this value suggests that a combination of both short and long-reads (*i.e.,* PacBio and/or Oxford Nanopore Technology) will likely be required for the assembly of a high-quality genome in this pathogen. The genome size estimation in-silico is not necessarily consistent with the wet-lab estimation (Leroy et al., 2007; Kikuchi, Akker & Jones, 2017). The concordance limitation between two methods is understandable since neither algorithms nor devices could comprehensively decode the complexity of nematodes genomes yet.

### Mitochondrial genome of *Hoplolaimus galeatus*

**Figure 1:**
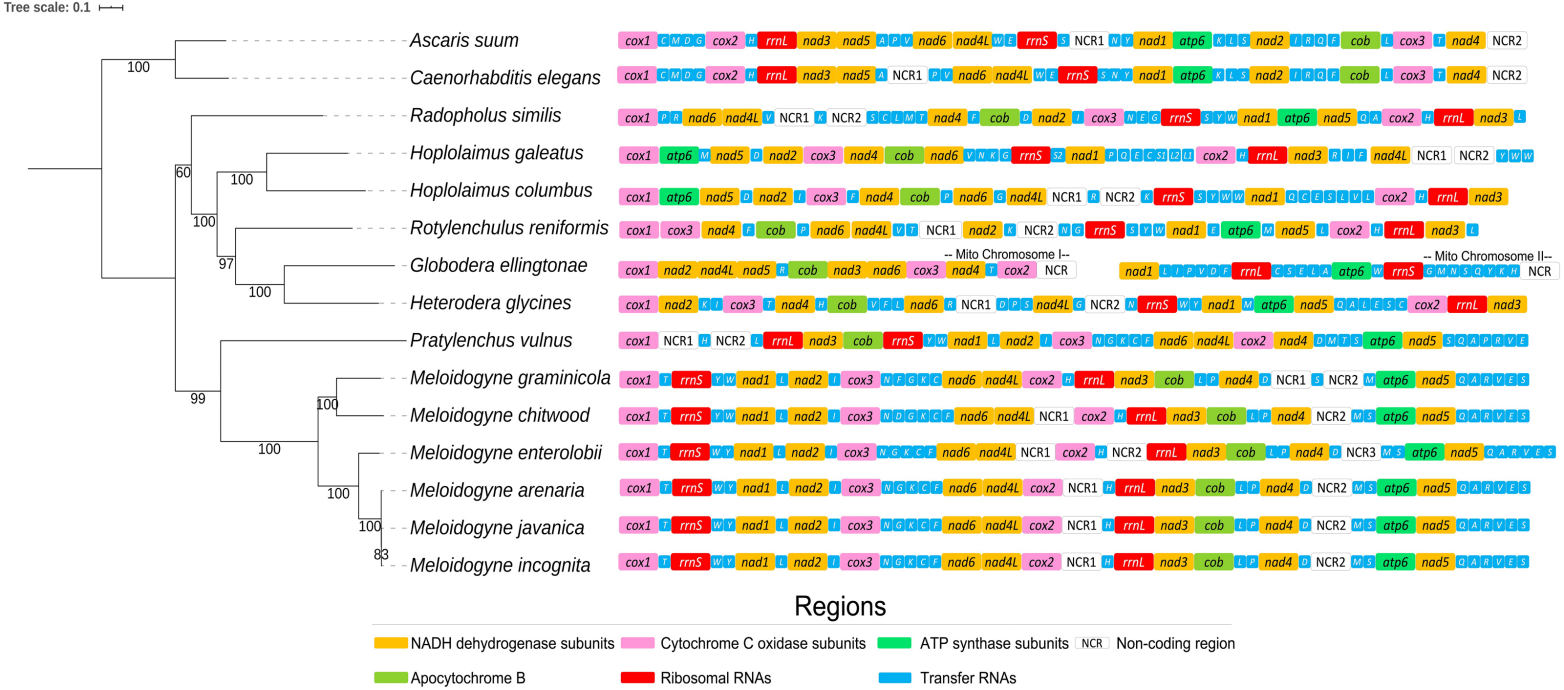
Phylogenetic position of *Hoplolaimus galeatus* and mitochondrial gene synteny in nematodes belonging to the class Chromodorea, superfamily Tylenchoidea. Phylogenetic tree obtained from Maximum Likelihood analysis was based on a concatenated alignment of nucleotides of the 12 protein-coding genes. In the analysis, *Caenorhabditis elegans* and *Ascaris suum* were used as the outgroup. Numbers below branches near nodes represent bootstrap values. See Methods and Results for further details.

The pipeline GetOrganelle assembled a nearly complete mitochondrial chromosome of *H. galeatus* 16,578 bp in length with an average coverage of 229x (GenBank accession number MK119781). The annotation with the pipeline MITOS2 indicated an assembly comprising 11 protein-coding genes (PCGs), two ribosomal RNA genes (rrnS (12S ribosomal RNA) and rrnL (16S ribosomal RNA)), and 16 transfer RNA (tRNA) genes. The PCG *atp8* is invariably missing in plant-parasitic nematode mitochondrial genomes (Ma et al., 2020). By contrast, the PCG *nad4l* is invariably present in all assembled mitochondrial genomes of plant-parasitic nematodes (Ma et al., 2020). tRNA genes found in the mitochondrial genome of the congeneric plant-parasitic nematode *H. columbus* but missing from the assembly in *H. galeatus* included tRNA-I, tRNA-F, tRNA-Y, and tRNA-W. All of the assembled PCGs and tRNA genes were encoded on the L-strand. The two ribosomal RNA genes were also encoded in the L-strand (Table 1). Various relatively long intergenic spaces involving > 260 bp in the mitochondrial genome of *H. galeatus* were observed. The gene order observed in the nearly complete mitochondrial genome of *H. galeatus* is quite different from that of the congeneric *H. columbus* (Fig. 1). In general, mitochondrial genome synteny is variable in nematodes, even within closely related species (Ma et al., 2020).

**Table 1:** Gene annotation and arrangement in the mitochondrial genome of *Hoplolaimus galeatus*.

**Name**	**Type**	**Start**	**Stop**	**Length**	**Start codon**	**Stop codon**	**Direction**	**Anticodon**	**Continuity**
*cox1*	Protein	1	1548	1548	ATT	TAA	Forward		12
*atp* 6	Protein	1561	2145	585	ATT	TAA	Forward		0
trnM	tRNA	2446	2215	70			Forward	CAT	8
*nad* 5	Protein	2224	3732	1509	ATT	TAA	Forward		-5
trnD	tRNA	3728	3785	58			Forward	CTG	44
*nad2*	Protein	3831	4601	771	ATT	TAA	Forward		45
*cox* 3	Protein	4646	5431	786	ATT	TAA	Forward		57
*nad4*	Protein	5489	6694	1206	ATT	TAA	Forward		58
*cob*	Protein	6753	7841	1089	ATT	TAA	Forward		51
*nad* 6	Protein	7893	8273	381	ATG	TAG	Forward		260
trnV	tRNA	8534	8590	57			Forward	TAC	1136
trnN	tRNA	9727	9783	57			Forward	GTT	91
trnK	tRNA	9875	9934	60			Forward	TTT	715
trnG	tRNA	10650	10705	56			Forward	TCC	344
rrnS	rRNA	11049	11619	570			Forward		-6
trnS2	tRNA	11614	11681	68			Forward	TGA	122
*nad* 1	Protein	11804	12694	891	ATA	TAA	Forward		-38
trnP	tRNA	12657	12710	54			Forward	TGG	2
trnQ	tRNA	12713	12765	53			Forward	TTG	562
trnE	tRNA	13328	13387	60			Forward	TTC	5
trnC	tRNA	13393	13445	53			Forward	GCA	3
trnS1	tRNA	13449	13506	58			Forward	TCT	59
trnL2	tRNA	13566	13620	55			Forward	TAA	63
trnL1	tRNA	13684	13738	55			Forward	TAG	38
*cox* 2	Protein	13777	14433	657	TTG	TAA	Forward		830
trnH	tRNA	15264	15317	54			Forward	GTG	358
rrnL	rRNA	15675	16163	489			Forward		26
*nad* 3	Protein	16190	16507	317	ATT	TAA	Forward		9
trnR	tRNA	16517	9	71			Forward	TCG	-9

### Phylogenetic placement of *Hoplolaimus galeatus*

The ML phylogenetic analysis (Fig. 1) confirmed the monophyly of the genus *Hoplolaimus* and the superfamily Tylenchoidea considering (i) the well supporter sister relationship between *H. galeatus* and *H. columbus* and (ii) the placement of the genus *Hoplolaimus* in a well-supported monophyletic clade together with *Radopholus similis*, *Rotylenchulus reniformis*, *Heterodera glycines* and *Globodera ellingtonae*. The aforementioned agrees with previous molecular phylogenies (Ma et al., 2020; Sun et al., 2014; Sultana et al., 2013; Humphreys-Pereira & Elling, 2014; Kim et al., 2015; Phillips et al., 2016). In the tree, all species belonging to the genus *Meloidogyne* clustered together into a well-supported monophyletic clade and *Pratylenchus vulnus* was recovered as a well supported sister clade to the genus *Meloidogyne*. The latter results also agree with (Ley & Blaxter, 2002) that recently suggested to classify Meloidogininae as a fully separate family based on the *SSU* rDNA phylogenies.

### Repetitive elements in the genome of *Hoplolaimus galeatus*

The pipeline RepeatExplorer identified a total of 299,509 clusters that comprised 97.7% of all analyzed reads (a sub-sample of 1,030,572 reads). The percentage of reads in the top 394 clusters that represent the most abundant repetitive elements in the genome of *H. galeatus* was 17%. A total of 274 repetitive elements families (clusters) comprising 106,693 reads were not assigned to known repeat families, and thus, were reported as ’unclassified’ by RepeatExplorer. The above agrees with the notion that studies focusing on the ’repeatome’ of *H. galeatus* and other plant-parasitic nematodes will likely result in the discovery of abundant new repetitive elements.

**Figure 2:**
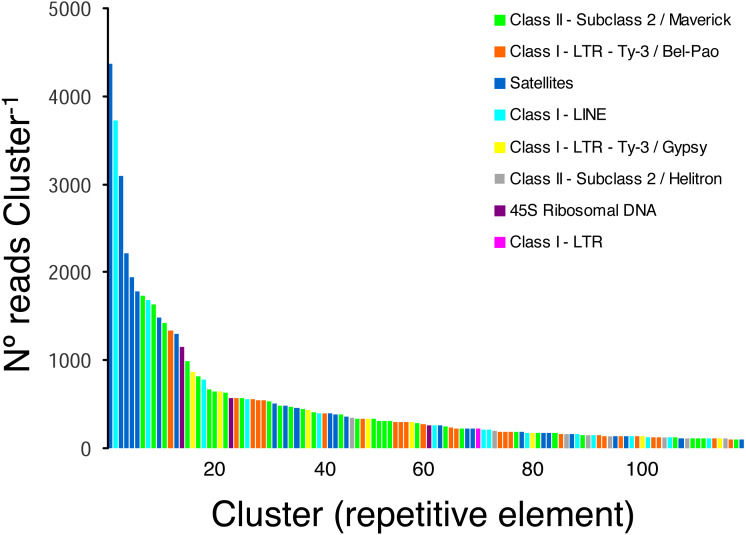
Frequency distribution and repeat composition of annotated clusters generated by similarity-based partitioning of in the lance nematode *Hoplolaimus galeatus*. Bars are colored according to the type of repeat present in the cluster, as determined by the similarity search in RepeatExplorer2.

Taking into account only clusters that were annotated by RepeatExplorer (*n* = 116 clusters), the most ubiquitous repetitive elements belonged to Class II- Subclass 2-Maverick elements (*n* = 32 clusters, 15,450 reads), Class I-LTR-Ty-3/Bel-Pao elements (*n* = 25 clusters, 7 674 reads), and satellite elements (*n* = 24 clusters, 20,637 reads), which were more abundant than Class I-LINE (*n* = 15 clusters, 8,804 reads), Class I-LTR-Ty-3/Gypsy elements (*n* = 8, 2,974 reads), and Class II-Subclass 2-Helitron elements (*n* = 8, 1,345 reads). Three clusters were classified as 45S ribosomal DNA (1,994 reads) and one cluster was classified as a Class I-LTR element (219 reads) (Fig. 2).

### Microsatellite discovery in *Hoplolaimus galeatus*

A total of 36 SSR primer pairs (*N* = 23 and 13 for 2mer and 3mer SSRs motifs, respectively) were identified using the most stringent filtering options for finding SSRs in pal_finder (Table 2). The software pal_finder did not report SSRs with 4mer, 5mer, and 6mer motifs.

Future studies combining mitochondrial PCGs or whole mitochondrial genomes (see above) and a subset of these newly identified SSRs (after further development) can be used to assess population genomic connectivity in *H. galeatus* from the southeastern USA.

## Discussion

Genome sizes varies considerably in nematodes, from ∼20 Mbp (*i.e.,* in plant-parasitic nematodes: 20.4 Mbp in *Hemicycliophora conida* [Hemicycliophoridae], 18.8 Mbp in *Pratylenchus coffeae* [Pratylenchidae] (Leroy et al., 2007), 74.6 Mbp in *Bursaphelenchus xylophilus* [Aphelenchoididae], 124.6 Mbp in *Globodera pallida* [Heteroderidae], 95.9 Mbp in *Globodera rostochiensis* [Heteroderidae], 96.7 Mbp in *Meloidogyne floridensis* [Meloidogynidae], 86.1 Mbp in *Meloidogyne incognita* [Meloidogynidae], and 53.0 Mbp in *Meloidogyne hapla* [Meloidogynidae] (Kikuchi, Akker & Jones, 2017)) to 270 Mbp in the pig roundworm *Ascaris suum* (Ascarididae) (Gregory et al., 2007; Moritz & Roth, 1976; Kumar et al., 2012).

**Table 2:** Simple sequence repeats (SSRs) in the genome of *Hoplolaimus galeatus* identified using the bioinformatic workflow Pal_finder v0.02.04.08 as implemented in the Galaxy platform ( https://palfinder.ls.manchester.ac.uk).

Forward primer name	Forward primer sequence	Reverse primer name	Reverse primer sequence	Motifs(bases)
galeatus_Forward_01	TGCATCTTGTACATGCCACG	galeatus_Reverse_01	GGCACGTTCAACAAGGACC	TGC(15)
galeatus_Forward_02	CAATATCAAAGAGAATTTTGATCACTACC	galeatus_Reverse_02	GGACCTCCCAGTTTCAATGC	ATT(15)
galeatus_Forward_03	AGAGGGCTGGAGAAACATGG	galeatus_Reverse_03	TTACGCTTTCGCTGTTCTCG	TCC(15)
galeatus_Forward_04	TCAAGCCACGGTACAACAGC	galeatus_Reverse_04	GCTCAAATTCCTCCATTCGC	AAC(24)
galeatus_Forward_05	ATTCCTTTTCTCAAAATTTCACG	galeatus_Reverse_05	TGTCATTGAGTACATCGGCG	ATT(15)
galeatus_Forward_06	TCCGGAAAATGTTTGCATCC	galeatus_Reverse_06	CCATTTGGAGTACACGCTCG	ACC(15)
galeatus_Forward_07	GCGATAGACGATCAAAGCCC	galeatus_Reverse_07	CCTTCAGTTCACGCACATCG	CCG(15)
galeatus_Forward_08	GAAGAACCATTTGGGGAGCC	galeatus_Reverse_08	AAAACAATGGTGGTCCGGC	AAT(15)
galeatus_Forward_09	GAGGGTTTTAGAGGGTGGGG	galeatus_Reverse_09	AGGGGTGAAGCAGGAGAACG	AAC(15)
galeatus_Forward_10	TCGTCGTTTTGTTTGTTCGG	galeatus_Reverse_10	GAAGGTACGGAAAGGGAGGG	TCC(15)
galeatus_Forward_11	ACAACGCCTCGACATCAGC	galeatus_Reverse_11	CGGATACCACCAGCCTCTAGC	CGG(15)
galeatus_Forward_12	GGCGAGATTTTCACTTTCTGC	galeatus_Reverse_12	GCATTCGGGACTATCCAACC	ATC(15)
galeatus_Forward_13	ATGGAGGATTACCAAGGCCC	galeatus_Reverse_13	GCGATATCTTCCCGTATGCC	AAC(15)
galeatus_Forward_14	TAGTTGGGCCGACTGACC	galeatus_Reverse_14	TCTTCTCTTCTGCCTCACCC	TC(14)
galeatus_Forward_15	CATATTTGGTGTGTTGGGGC	galeatus_Reverse_15	AAATCTATCCGCACTTTTCCG	TC(12)
galeatus_Forward_16	GGGAATGAGTGCTCCAAAGC	galeatus_Reverse_16	CGTATTCGAATTCATGCACCC	TC(12)
galeatus_Forward_17	ACCCATTCATTTCTCTCGCC	galeatus_Reverse_17	TGGCCAAGTCTTTCTCTCCC	TC(12)
galeatus_Forward_18	GAATGACAGAGAGAGGCAGGG	galeatus_Reverse_18	TCACTTGCTCTCTGAATTTCTTGG	TC(14)
galeatus_Forward_19	GAAGATAGTGAGAGACTGAGAAATGG	galeatus_Reverse_19	CTCGCTTTCCTCTTCCTGC	TC(22)
galeatus_Forward_20	CAAACCAATTGTAATCAGATGATCC	galeatus_Reverse_20	AAACAGTCAAATGGCTGGGG	TC(12)
galeatus_Forward_21	TTTCCAAAACCTCTGGTGCC	galeatus_Reverse_21	AGAATTACCGAATCGCGACC	TG(14)
galeatus_Forward_22	TTCTTACCCTCTGCGCTTCG	galeatus_Reverse_22	CAATCCTCAGCACTCCCACC	TG(12)
galeatus_Forward_23	GTTCGAGATCAGCTGGCAGG	galeatus_Reverse_23	AACTAGCCCCTGGCACACC	CG(12)
galeatus_Forward_24	ATCTCCGGATTCAAAGCAGC	galeatus_Reverse_24	AAATTCGCAATGAGCATCCC	TC(16)
galeatus_Forward_25	CGTTTGGAAGGTTCATTTCAGC	galeatus_Reverse_25	TCGGGGTTGTAGGAGTTTGG	TC(12)
galeatus_Forward_26	TTCTTCAGCGTTCATCTCCG	galeatus_Reverse_26	GGGATGATGAGTAAAGCGGC	TC(44)
galeatus_Forward_27	GAGAGAGAAAGACGGAGCGG	galeatus_Reverse_27	AGTGCCCAATACATGAGCCC	TC(14)
galeatus_Forward_28	ATGCGGATTCTCTGGCTCC	galeatus_Reverse_28	AAGACAAGTGATCCAGCAGACG	TC(14)
galeatus_Forward_29	CTCTGACTGTATGCCGTCGC	galeatus_Reverse_29	GTGAAAATGAGAGATGGCCG	TC(12)
galeatus_Forward_30	AGGACGACATAATGGGTCGG	galeatus_Reverse_30	TCTTCCTCCAGCTAGCAGCC	TC(14)
galeatus_Forward_31	TCGCTCTATCTCTCGTGCCC	galeatus_Reverse_31	ACATAATATCGCTCACACGATGC	TC(14)
galeatus_Forward_32	GAAGAAGGGGTGGGAATGG	galeatus_Reverse_32	ACGACATGTGCGTTTTGTCC	TC(16)
galeatus_Forward_33	CCAGCCACTACCAGGAGACC	galeatus_Reverse_33	GATGAATAACTCGCGCACCC	TC(12)
galeatus_Forward_34	TTGGCCTGTCTTCTATTTCACC	galeatus_Reverse_34	TCATTACACAACGTGGCCG	TC(12)
galeatus_Forward_35	CTCCTTGTCCCTGCCTATGG	galeatus_Reverse_35	GGCGCTGCTTACACTTATTGC	TG(12)
galeatus_Forward_36	AACCTTTCTCTTATACACATTTTCTATCC	galeatus_Reverse_36	TGACTATTAAACACATCTAATGCTACCG	TG(12)

In the Nematoda, repetitive elements have been characterized in-depth in the model species *Caenorhabditis elegans* and a few of its closest relatives (Malik, Henikoff & Eickbush, 2000; Bessereau, 2006; Kozlowski et al., 2020; Phan et al., 2020) and most recently in non-model species such as the rice root-knot nematode *Meloidogyne graminicola* (Phan et al., 2020). Although the number of published nematode draft genomes has increased steadily during the last years, the ’repeatome’ is poorly studied in most nematodes. Taking into account only annotated repeats, this study revealed that a large part of the repeats in the genome of *H. galeatus* represent various families of Class II- Subclass 2-Maverick elements, LTR elements, and satellite DNA. Repetitive elements have been shown to account for a relatively small (12% in the genome of *Caenorhabditis elegans*) (Bessereau, 2006) or large (32% in the soybean cyst nematode *Heterodera glycines*) (Masonbrink et al., 2019) portion of the genome in the nematodes in which the genomic ’dark matter’ has been explored (Bessereau, 2006). Given the large number of repeat families not annotated in this study, our results further suggest that details studied focusing on repetitive elements of non-model nematodes will likely result in the discovery of a considerably number of new repetitive elements.

## Conclusions

This study developed genomic resources for the plant-parasitic nematode *Hoplolaimus galeatus*. Using low-pass short read Illumina sequencing, the genome size was estimated in-silico, a nearly complete mitochondrial chromosome was assembled, and nuclear repetitive elements were identified, partially classified, and quantified. A set of SSRs was also detected. This information will contribute to the better understanding of meta-population connectivity and putative genomic mechanisms involved in the exploitation of the broad range of host plants used by *H. galeatus*.

## Additional Information and Declarations

### Data Availability

The following information was supplied regarding data availability: A total of 12,592,874 paired-end (PE) reads (300 bp) were generated and are available in the Sequence Read Archive (SRA) repository: PRJNA659265, SAMN15902603, SRR12516298.
